# A Life in Passing: Jonathan Gray

**DOI:** 10.1186/1479-5876-5-54

**Published:** 2007-10-30

**Authors:** Michael T Lotze, Jonathan Gray

**Affiliations:** 1 Departments of Surgery and Bioengineering, University of Pittsburgh Cancer Institute, Rm G.27 Hillman Cancer Center, 5117 Centre Avenue, Pittsburgh, PA 15213 USA

## 

Jonathan Gray died yesterday. Five years ago, he underwent an esophageal resection for cancer, developed an aorto-esophageal fistula requiring an emergent colonic interposition and survived to develop metastatic disease to the liver, initially successfully treated with oxaliplatin and a taxane containing regimen. Gifted with a sense of life's meaning and purpose, a rather remarkable ability to single out the best and brightest most capable of helping him, he was a tireless enthusiast for making novel therapies available to patients, and rallied against the futility of the current drug development process – too slow, too ineffective, and as he faced his own certain and early mortality, impossibly oriented for the patient with cancer to limited success for a limited number of individuals with extraordinarily slow mechanisms.

Believing that an immune response was his best hope for success, he visited and encouraged the tumor immunologists in the world that he felt were most capable of helping him. I find myself fortunate in being one of those. In spite of the extraordinary demands of his treatments over the last few years, he persisted in making appeals to his friends and colleagues to press the case for the urgency of cancer research and rapid application of novel therapies. With pain from a celiac plexus mass, a fungal infection in trapped lung requiring multiple hospitalizations, and diffuse metastatic hepatic disease, he rallied others to find new strategies for cancer. For all of us who Jonathan consulted, we are diminished by his passing, and furthermore chastened to do more, imagine more, find more and apply to others like him. He deserves no less.

All of us who work in the fields of cancer research and therapy should applaud his vision, his magnanimity, and devotion to what we hope will make the future scientists and clinicians stand erect to take on the challenge of the disease that ultimately took his life. We can indeed do more, imagine more, find more and apply. Jonathan, bless you and rest in peace. We are driven by your example and your extraordinary hope. We too, like his remarkable supportive family, are saddened by his passing, but pass on his thoughtful reflections [with only modest editing] on the needs he perceived but woefully too slow in responding to his need to see his sons futures realized and live for his parents and loved ones, just shy of his 60^th ^birthday. Jonathan, you live on in our hearts and dedication to delivering better therapies, more expeditiously, and more effectively. Thank you for being there when we needed you and for projecting your concerns on a receptive oncologic audience. We are working now for others; and of course want your just repose.

## It's Cancer

Jonathan E. Gray

Manhattan, New York City, USA

Approximately 50% of all American men and 33% of all women alive today are expected to be diagnosed with cancer, and just under 50% to die of their disease. This is the current view of scientists and clinicians, and unlike the situation in cardiac disease, in which halving of mortality for those dying under the age of 85 has been realized, cancer therapy has only enjoyed minimal success and is now the leading cause of death for those individuals.

Cancer is something that older people get, and I can escape with a healthy diet, exercise and not smoking. Besides, there's a war against cancer, and probably hundreds of billions of dollars and thousands of geniuses doing research to cure the disease, with progress so rapid they'll have it cured by the time I'm fifty. This may approximate the more common, if subliminal, attitude.

Is the correct view one of optimism, or one of skepticism and anger? This is a question on which experts will disagree, and I am not an expert. I am a cancer patient. Yet, even without exhaustive study, certain questions command immediate attention.

To begin with the obvious comparison, in 1941 in response to a letter from Einstein, FDR created "The Manhattan Project," sparing no resources, and within thirty months (and $20 billion in ***today's ***dollars) we detonated the first atomic bomb.

In 1971, Nixon declared a "War on Cancer," with the objective of finding a cure in five years. Thirty-five years later, while enormous strides have been made in basic biological science and technology, the actual "battle statistics" are not that encouraging. There has been dramatic success against pediatric cancer and blood malignancies, but only meager success in treating solid tumors in adults, of the breast, lung, colon, liver, kidney, pancreas, esophagus and so forth. Early detection and surgery remain the only hope for survival against these tumors. There are no cures once the disease has metastasized, except in the rare instance of melanoma and renal cell carcinoma in patients treated with Interleukin 2.

Approximately 1.9 million Americans are diagnosed with some form of cancer and 600,000 die of the disease annually. Aside from smoking related cancers, the incidence has remained fairly stable on an age-adjusted basis for decades.

Except for not smoking, the notion that by healthy living one has significant control over the risk is exaggerated. Cancer is a programming error, a software glitch in one's genes. Mistakes, damage, mutations... arise ***naturally ***due to copying errors when cells replicate.

Cells grow, divide and multiply. When the cell divides, it copies its DNA so that each of the two resulting cells gets an identical copy. These DNA molecules are millions of molecules long, and consist of all the instructions for the cell to function.

Now, while this copying is highly precise, with only about 1 molecule in a billion a mistake, we consist of hundreds of trillions of cells, and over time, mutations accumulate. And yet, it takes the unlikely occurrence of several mutations in the same cell, generally 4 – 7, for it to become malignant, dividing without cessation, traveling throughout the body and invading normal organs instead of staying where it belongs. All it takes is one cell that evades the surgeon's knife, or the radiation, or one's immune system, and it's over. By the time the earliest cancers are detected, at just 1 cm, no bigger than an orange seed, they already consist of a hundred million malignant cells.

Cancer is a limit that nature has placed on a species whose lifespan has increased! It's a disease that arises from a failure of your genes to control the behavior of your cells.

The analogy is made with a car whose gas-pedal is stuck on the floor and it's just racing. Thank goodness for the brakes! Now suppose the brake pedal gets stuck, and cannot be depressed to stop the car, and the emergency brakes and steering wheel fail. The threshold of 4–7 errors has been reached, and the car is out of control.

The tremendous strides that have been made in basic science are not readily applied to patients. Frequently physicians are behind in their knowledge of the science, while the scientists do not deal with patients. They deal with mice. With all the hoopla over biotherapeutics, try to find an immunologist to consult with your oncologist concerning their use.

Most if not all tumor cells have special molecules on their surface which identify the particular genes that have failed. We should have been building a national data bank, saving frozen pieces of tumors, testing them and categorizing them into subsets based on their particular tumor markers, and then checking which subsets of various tumors respond to which therapies. Instead, tumor tissue is rarely frozen, tested and the information saved and studied. It's discarded as garbage! Or, the information isn't shared between institutions, or there are software incompatibilities, non-standardization of reagents, or, it is guarded as intellectual property.

One can read of clinical trials for a new drug that targets a particular marker, and even if your tumor bears the same molecular target, you may not be permitted that drug. Why? Because tumors are still categorized by their organ of origin. If the drug is used to treat patients with melanoma, never mind that your gastric tumor has the same molecules, it's stomach cancer, not skin cancer.

At any point in time there are hundreds of thousands of terminal cancer patients, with 3 months to live. Yet there are always many experimental drugs that have passed phase I and II tests for toxicity, and we know have no horrible side effects. We just don't know how effective they'll be, if at all. Well every one of these patients would give anything for some hope, for a chance at an experimental drug, particularly if the downside-risk were a skin-rash, or diarrhea.

Moreover, scientists complain that research that works in mice doesn't always work in humans. Yet, here's a chance to work with human subjects where risks are manageable. However, the drug companies are understandably reluctant to provide experimental use of these drugs because the liability attorneys are waiting to pounce on them. Even if the patient signs a letter absolving everyone of liability, the courts may disregard it. ***The Congress should address this situation immediately! ***Indeed, it may constitute a violation of the 14^th ^Amendment, as a deprivation of life without due process of law.

Twenty five years ago, policy makers were upset that 10% of US GDP was spent on healthcare, versus 6% in Europe. So they decided to run healthcare like a business, with HMOs, hospitals and insurance companies all squeezing one another for profits. It was supposed to be more competitive and efficient. Result? We now spend 15% of GDP on our healthcare system, which ranks the US number 29^th ^in the world, between Slovenia and Portugal, with 40% of the costs estimated to be duplicated administrative expense.

Gifted scientists may spend one third of their time writing grant proposals for the NCI instead of doing research.

I never could understand our national concern with healthcare spending as a percent of our economy, provided it wasn't being wasted. The economy is simply what we choose to do from 9 to 5 each day. Is it really preferable to produce discretionary consumer goods, toys, than to invest in our health? Many countries have healthy life-spans that are 4 – 5 years longer than ours. Is it really rational to prefer a consumer electronics device?

Why is a graduate in molecular biology offered four times as much money to work on Wall Street as in a research lab? Why is a potential research break-through, on which thousands of lives may depend, delayed a year due to lack of funding for an inexpensive technician? Why does an Assistant Professor at a prestigious medical school earn $60,000 while an NFL wide receiver earns $9,000,000?

These are not the kind of decisions Adam Smith believed would be made optimally by "an invisible hand." The system breaks down because of a lack of information on the part of the voting public, and a culture geared toward the production of consumers. And apparently, to some, it doesn't really matter what we consume.

What would have happened if in 1941 FDR had given the Manhattan Project to the free market and allowed hundreds of companies to compete on a for-profit basis to develop the bomb, and not to share information? We'd be speaking German.

For Smith capitalism worked because market participants could understand their choices and they had a choice between competing goods that could be substituted for one another. How many of us are equipped to discern whether Genentech's Avastin or Pfizer's Sutent are better, or helpful at all. As the ads relentlessly repeat, "Why not ask your doctor if arsenic trioxide is right for you?" Unfortunately many doctors have spent little time studying, and biology has changed profoundly in the past 15 years.

One chemotherapeutic drug may add 3 months, another 5 months to survival that would be 15 months untreated, and with virtually no prospect of a cure. There is enormous revenue to be gained from market share, and always at a meaningful increase in price. Is this really understood by the public?

Cancer drugs are tested frequently as single agents, and must be shown to be effective as such to be approved by the FDA. Yet cancer is known to arise from multiple genetic errors. Can multiple failures be repaired with only one treatment mechanism? If we only got the steering wheel fixed in that car, would the passengers have survived at high speed with no means to stop? Would it make more sense to test cancer drugs in combinations, therapeutic cocktails, as has worked in AIDs? If so, how many drugs that would have been effective in combination with other drugs have instead been discarded over the past 35 years?

There are times when the war on cancer resembles more of a traffic jam than a war. One is tempted to call for a Manhattan Project in the war against cancer. Unfortunately, there is little confidence in government, which would likely just produce a more spectacular traffic jam. It has been suggested that such a public effort on a grand scale be directed by a politically independent agency, something like the Federal Reserve Board, composed of top scientists managers; perhaps we should create it.

In fact, the wealthiest amongst us, the top 1/2 of one percent, literally have a surplus of money. They should, according to Adam Smith, demand higher quality goods, like a cure for breast cancer or prostate cancer, and added years of life. However, if this can best be done with government intervention, they should lobby Congress to impose a tax, so as to yield an incremental $15 billion or so annually, initially, and increase this amount progressively to perhaps $60 billion or more within a few years. Ironically, real progress at reducing cancer would reduce the total expenditures on health care and add meaningfully to the economy no matter how one measured wealth.

So life goes on. You live a healthy, happy life for 45 – 65 years, and then it happens, the trap door opens beneath you and you fall into a Kafkaesque, nether world, where ultimately the patient is responsible for making a set of choices that are dauntingly complex, expensive, and upon which his life depends, while simultaneously dealing with major surgery, chemotherapy and radiation, with nausea, profound fatigue, and impaired mental focus.

The total expenditure on cancer research in the US is approximately $15 billion annually, within the context of a $1.8 trillion budget for government at all levels. This includes just $4.75 billion at the NCI in 2007. Noncommercial funding organisations in Europe collectively spent $1.43 billion on cancer research 2002–2003. This ranged from $388 million in the United Kingdom to $0 in Malta, with only  three countries spending greater than $100 million. In Euros the 3.6 billion for the US NCI is more than two and a half times the 1.43 billion expended by  European noncommercial sources. The EU spends relatively more on cancer biology than does the US (41% compared with 25%). The US spends a greater proportion of its cancer research funding on research into prevention and treatment than does the EU (prevention, 9% in the US compared with 4% in the EU; treatment, 25% in the US compared with 20% in the EU). In the US, the funding, which has flattened in the last three years, is roughly equivalent to what is spent annually on pet supplies and car washes combined. Investment in cancer research is just $51 per capita, as compared to per capita income of $33,000. We still spend $290 per capita on tobacco products, and for those who are curious, $1,750 on defense.

Within the next decade, the average age of the U.S. population will increase and lead to a large increase in annual cancer incidence, perhaps 500,000 on top of the current 1.9 million cases, or 2.4 million, with annual deaths rising 250,000 to $850,000. Baby boomers, beware!

We will all be victims of cancer, whether as patients, or as spectators to its destruction of those we love.

According to Leroy Hood of the Institute for Systems Biology, significant progress against cancer may extend healthy longevity by ten to thirty years. If the manufacture of a car is measured in tens of thousands of dollars when calculating GDP, how do we value life itself? Your mother's, your husbands, your friend's, your own?

$51 per person per year toward cancer research out of $33,000 in total per capita income! One sixth the price of an iPod. A dramatic investment in cancer research and clinical care seems urgently needed and long past due. It is of secondary importance, but likely nonetheless, that such an investment would bring handsome returns in conventional economic terms as well. And finally, pray let this cause be taken up in the interest of the public good of all humanity, and not become a petty partisan squabble.

**Note**: Jonathan E. Gray was a securities analyst on Wall Street with the firm of Sanford C. Bernstein & Co., specializing in research on financial companies for over thirty years, until retirement for medical reasons. His demise now gives us pause. Think again.

